# Meta-analysis of the effects of physical activity on executive function in children and adolescents with attention deficit hyperactivity disorder

**DOI:** 10.1371/journal.pone.0289732

**Published:** 2023-08-17

**Authors:** Yiling Song, Biyao Fan, Chunshun Wang, Hongjun Yu

**Affiliations:** 1 Department of Physical Education, Tsinghua University, Beijing, China; 2 College of Physical Education and Sports, Beijing Normal University, Beijing, China; University of Illinois at Urbana-Champaign, UNITED STATES

## Abstract

**Background:**

Executive function is a core deficit in children with attention deficit hyperactivity disorder (ADHD). This study systematically reviewed the evidence for the effects of physical activity (PA) interventions on executive function in children and adolescents with ADHD and explored the moderating effects of key variables of PA on executive function.

**Methods:**

Relevant literature in four electronic databases, Pubmed, Web of Science, Cochrane Library, and Embase, were systematically searched. Revman 5.4 was used for data analysis, and combined effect sizes, heterogeneity tests, subgroup analyses, and sensitivity analyses were calculated. Egger’s test in Stata 15.0 was used for publication bias testing.

**Results:**

A total of 24 articles with 914 participants were included. Meta-analysis showed that PA interventions improved inhibitory control (SMD = -0.50, 95%CI [-0.71, -0.29], P < 0.00001), working memory (SMD = -0.50, 95%CI [-0.83, -0.16], P = 0.004) and cognitive flexibility in children and adolescents with ADHD (SMD = -0.45, 95%CI [-0.81, -0.09], P = 0.01). Subgroup analysis revealed a moderating effect of intervention intensity, motor skill type, sessions of PA, and weekly exercise volume on executive function.

**Conclusion:**

PA interventions had positive effects on improvements in core executive functions in children and adolescents with ADHD and were influenced by intervention intensity, type of motor skill, sessions of PA, and amount of exercise. This has practical implications for the formulation of PA interventions programs.

## Introduction

Attention-deficit / hyperactivity disorder (ADHD) is childhood’s most common neurodevelopmental disorder [1]. Its core symptoms are inattention, hyperactivity, and/or impulsivity [2], and are often accompanied by cognitive impairment and learning difficulties [3, 4]. Studies have shown that the global prevalence of ADHD is about 7.2% and the incidence is still increasing [5]. ADHD tends to last into adulthood and is a risk for other mental health disorders and adverse outcomes, including poor academic achievement, employment and relationship difficulties, and delinquency [6].

Executive functions are a set of interconnected cognitive skills that are required to learn, cope, and manage daily life. It is generally agreed that three core executive function components exist: inhibition control, working memory, and cognitive flexibility [7]. Effective executive functions are essential for promoting healthy childhood development because they support cognitive, social, and psychological growth and can help predict academic and life success [7–9]. Studies have found that impaired executive function is one of the core deficits of ADHD [10, 11], and children with ADHD have varying degrees of impairment in inhibitory control, working memory, and cognitive flexibility [12–15], which seriously affects the quality of life, behavior management, social skills, and academic performance of children with ADHD, and has a negative impact on their physical and mental health [16, 17].

At present, pharmacotherapy is one of the main treatments for ADHD [18]. However, researchers have found that long-term medication can increase patients’ drug tolerance [19]. In addition, some children with ADHD experience adverse effects such as decreased appetite, insomnia, headache, and nausea [20–22], leading to poor adherence [23]. Behavioral and psychological interventions are common non-pharmacological treatments for ADHD [24–26], and although they have no side effects, they are too cumbersome and slow to be effective, making it difficult to maintain continuous treatment [27]. There is an urgent need for alternative or adjunctive means of intervention treatment for children with ADHD that are operable, effective, and highly adherent with few side effects. In recent years, researchers have found that physical activity(PA) interventions can improve ADHD children and adolescents’ attention, cognitive impairment, social behavior, and motor performance [28–30]. Therefore, as a new non-pharmacological therapy, PA interventions have received extensive attention from researchers.

Recent studies have found that PA interventions can improve the executive function of ADHD patients [31]. For example, Welsch et al. found that chronic PA interventions had positive effects on inhibitory control, cognitive flexibility, and working memory in children with ADHD compared with untreated controls, but the authors did not examine the effects of different types, intensities, and frequencies of PA interventions on improvements in executive function [32]. Several researchers have systematically reviewed the evidence for the effects of PA on cognition and behavior in children and adults with ADHD, and the majority of results from included studies suggest that acute and chronic PA interventions can improve cognition and behavior in people with ADHD, but this study only systematically reviewed the results of previous studies and did not perform a quantitative combined analysis [33]. In addition, a previous systematic review reviewing only the effects of acute PA interventions on young people with ADHD showed improvements in symptoms during exercise in children with ADHD compared to other sedentary tasks such as watching videos, and found that 5 minutes of jumping or 30 minutes on a treadmill or stationary bike was sufficient to significantly improve inhibitory control or cognitive and executive function [34]. However, Verret et al. found no significant improvement in response inhibition in children with ADHD after a single 30-minute session of moderate to vigorous aerobic exercise [35]. A study by Piepmeier et al. showed that a moderate to vigorous physical activity (MVPA) intervention significantly improved inhibitory control in children with ADHD, but their working memory and cognitive flexibility did not change significantly [36]. Thus, the current results on the effects of PA interventions on executive function in children with ADHD are mixed and need to be further explored. Furthermore, Seiffer et al. only systematically analyzed the efficacy of regular MVPA in children with ADHD and concluded that MVPA could be used as an alternative treatment for ADHD, but the efficacy of different intensities of PA in children with ADHD was not explored [37].

Despite the work mentioned above, three major gaps in the scientific literature still need to be addressed. First, there is a lack of research on the effects of key moderating variables of PA(e.g., intensity, type, and frequency of PA) on three core executive functions (inhibitory control, working memory, and cognitive flexibility) in children and adolescents with ADHD. Second, although some studies have shown positive effects of PA interventions on improving executive function in individuals with ADHD, each study used a different intervention protocol, which produced different intervention effects on executive function. The dose-effect relationship between PA interventions and executive function still needs further investigation. Third, most current systematic reviews have primarily analyzed the effects of PA interventions on overall executive function in children with ADHD. However, few meta-analyses have systematically examined the effects of PA on the three core executive functions in children with ADHD. Since motor interventions may only affect a specific executive function domain, a more detailed analysis is done.

The purpose of this meta-analysis was to conduct a systematic quantitative analysis of studies on the effects of PA interventions on three core executive functions (inhibitory control, working memory, and cognitive flexibility) in children and adolescents with ADHD. The secondary purpose was to examine the effects of moderating factors (intensity, type, frequency, and duration of PA) on inhibitory control, working memory, and cognitive flexibility in adolescents with ADHD. In order to provide a theoretical basis for the development of rational PA interventions programs for children and adolescents with ADHD.

## Methods

This meta-analysis followed the recommendations provided by the Preferred Reporting Items for Systematic Reviews and Meta-Analysis statement [38]. And the meta-analysis is registered with PROSPERO (registration number: CRD42023388409; https://www.crd.york.ac.uk/PROSPERO/).

### Literature search

A comprehensive literature search of four electronic literature databases (Web of Science, Pubmed, Cochrane Library, Embase) was conducted simultaneously. The search strategy was based on a combination of subject terms and free terms. It was determined after repeated pre-checks and supplemented with manual searches, retroactively including references to the literature when necessary. The search period was from the beginning of each database to March 2023. Take PubMed as an example, and the specific search strategy is shown in Table 1.

**Table 1:** Pubmed search policy.

Serial No.	Search contents
#1	sport* OR exercis* OR physical activit* OR physical exercise OR physical education OR acute exercise OR chronic exercise OR aerobic exercise OR resistance exercise OR exercise intervention OR exergaming OR fitness
#2	ADHD OR attention deficit hyperactivity disorder OR attention deficit hyperactivity disorders OR attention deficit disorder with hyperactivity OR attention deficit disorder OR hyperactivity disorder
#3	child* OR school age OR youth preschool OR preschoolers OR adolescen* OR teenage* OR youth
#4	cognitive function OR cognition OR cognitive benefits OR cognitive performance OR executive function OR inhibition OR inhibitory control OR response inhibition OR working memory OR memory OR shifting OR cognitive flexibility OR cognitive control OR shift cognitive OR updating OR planning OR processing speed
#5	#1 and #2 and #3 and #4

### Inclusion and exclusion criteria

The inclusion criteria were as follows: (1) participants were children and adolescents with ADHD <18 years old [39]; (2) participants must be diagnosed with ADHD by the psychiatric physician or meet the requirements listed on validated rating scales (e.g., DSM-IV, DSM-V, ICD-10, SNAP-IV); (3) studies must examine the effects of PA, exercise, and physical exercise on executive function in children and adolescents with ADHD; (4) outcome indicators include executive function, inhibitory control, working memory, and cognitive flexibility; (5) studies included randomized controlled trials, crossover or parallel group comparison trials; (6) papers published in English.

The exclusion criteria were as follows: (1) participants had dyskinesia that prevented intervention; (2) the experimental group was a combined intervention study; (3) repeatedly published literature with poor quality assessment; (4) case studies, review literature, conference papers, and dissertations; (5) non-English papers.

### Study selection

The two researchers screened the literature independently according to the inclusion and exclusion criteria. First, the papers were initially screened by reading the title and abstract. Second, the full text was downloaded after obtaining the eligible literature, and full text screening was performed. Finally, the two researchers compared the independently screened literature. For the literature with inconsistent screening results, the decision to include or not was to be made by a joint discussion with the third researcher.

### Data extraction

Information related to the included literature was extracted independently by two researchers using a standardized form. The extracted information was as follows: (1) basic information: authors, year of publication, and nationality of the authors; (2) basic characteristics of the participants: ADHD diagnostic criteria, age, sample size, etc.; (3) PA interventions protocols: intervention period, type, duration, intensity, frequency, etc.; (4) how the outcome indicators were assessed; and (5) indicators of the quality evaluation of the literature: method of generating the random sequence, concealment of the sample allocation sequence, blinded method implementation, completeness of the outcome indicators, and the availability of selective reporting of results.

### Risk of bias assessment

The risk of bias in the literature was assessed using the Cochrane Collaboration tools. The evaluation criteria included: random sequence generation, allocation of hidden protocols, blinding of study subjects and intervention implementers and outcome assessors, completeness of outcome indicators, selective reporting of results, and other potential risks of bias. The risk of bias was judged according to specific evaluation criteria, with "yes" representing a low risk of bias, "unclear" representing a lack of relevant information or uncertainty of bias, and "no" representing a high risk of bias. The assessment of the risk of bias was performed by two evaluators independently and verified each other, and in case of disagreement, a third evaluator made the decision.

### Data analysis and synthesis

Data analysis was performed using Reviewer Manager 5.4 software to calculate combined effect sizes, heterogeneity tests, subgroup analysis, and sensitivity analysis. Heterogeneity was tested using *p*-value and I^2^. If P < 0.05 and I^2^ > 50%, it indicated a significant heterogeneity and a random-effects model could be used for meta-analysis; otherwise, a fixed-effects model was used. The continuous outcomes were expressed as standardized mean difference (SMD) and its 95% confidence interval (CI). Sensitivity analyses were performed by changing the statistical model and excluding literature one by one. Egger’s test in Stata 15.0 software was used for publication bias testing. If publication bias was present, the trim and fill method was used to correct for publication bias.

## Results

### Search results

**Fig 1:**
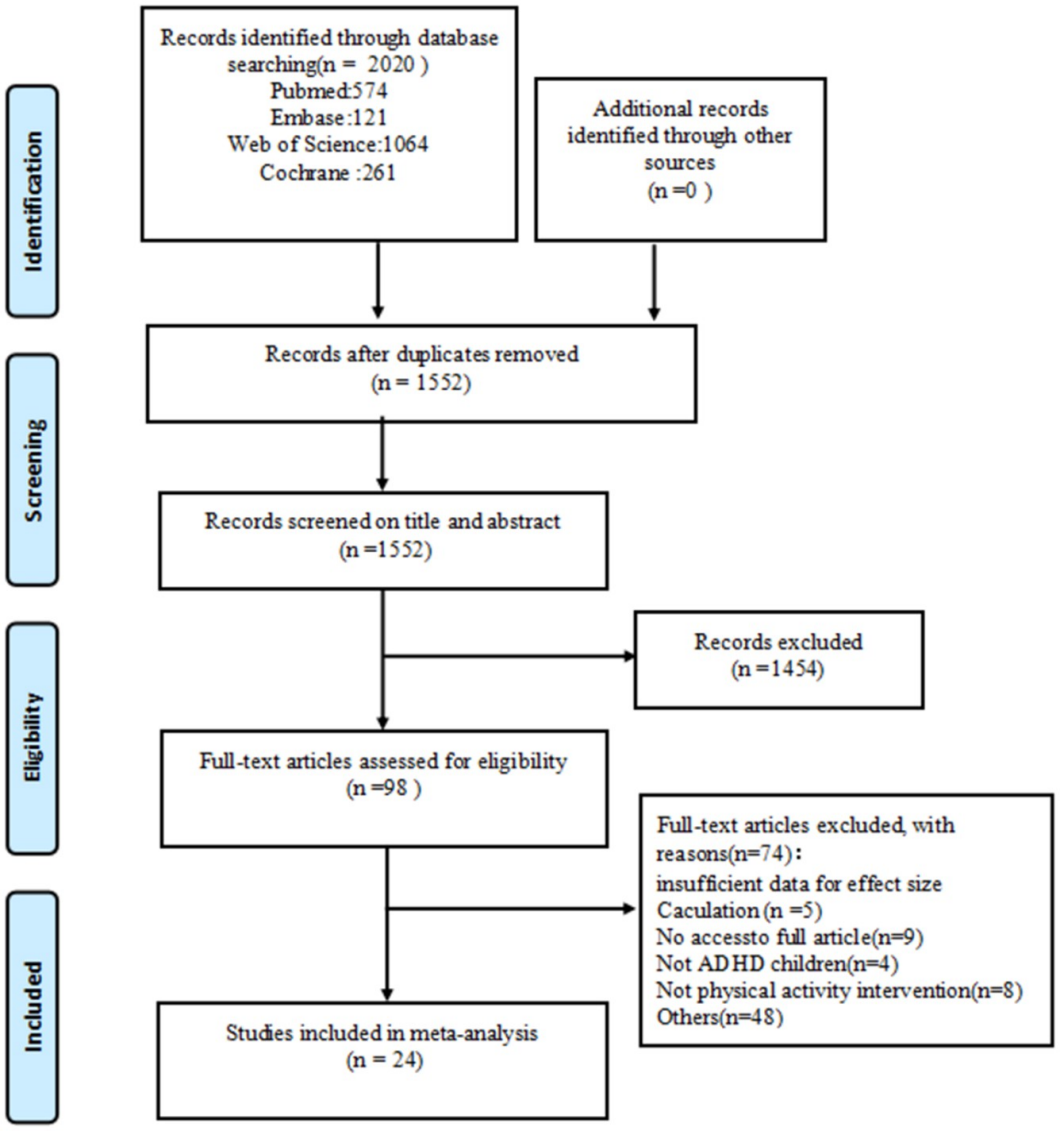
Flow diagram of study selection.

A total of 2020 articles were retrieved from the database, and 1552 were obtained after excluding duplicates. The 1454 articles that were irrelevant to this study were removed by reading the titles and abstracts, and 98 were obtained. After further full-text screening, 74 articles were excluded, and 24 were included in the meta-analysis. The specific screening process of the literature is shown in Fig 1.

### Basic characteristics and risk bias of the included studies

As shown in Table 2, 24 studies with a cumulative sample of 914 ADHD were included. 10 of the included studies were MPA interventions and 6 were MVPA interventions; the types of PA interventions used included 13 closed-motor skills and 12 open-motor skills; the frequency of chronic PA interventions in the ADHD experimental group was generally 2–3 times/week, and the intervention period was generally 8–12 weeks; the ADHD control group often used watching videos or maintaining regular activities as a control.

**Table 2:** Basic characteristics of included studies.

Study (year)	Study design	Participant description		Intervention							Control condition	Outcome Measures
		Sample Size (IG/CG)	Age (years)	Content	Intensity	Duration (min)	Frequency (times/week)	Week	Closed/Open motor skills	Acute/ Chronic		
Benzing et al. (2018)	RCT	24/22	8–12	Exergaming	MVPA	15	-	-	Open	Acute	Watching video	Flanker Task,CSB
Benzing & Schmidt (2019)	RCT	28/23	8–12	Exergaming	NR	30	3	8	Open	Chronic	Watching video	Simon Task;Flanker task; Color span backward
Bigelow et al.(2021)	Crossover	16/16	10–14	Cycling	MPA	10	-	-	Closed	Acute	Reading	Stroop Task;The Leiter-3 Reverse Memory;TMT
Chang et al.(2012)	RCT	20/20	8–13	Running	MPA	30	-	-	Closed	Acute	Watching video	Stroop Test;WCST
Chang et al.(2014)	nRCT	14/13	5–10	Aquatic Exercise	MPA	90	2	8	Open	Chronic	NR	Go/No Go Task
Chuang et al.(2015)	RCO	19/19	8–12	Running	MPA	30	-	-	Closed	Acute	Watching video	Go/No Go Task
Da Silva et al. (2019)	RCT	10/10	11–14	Swimming	MPA	45	2	8	Closed	Chronic	NR	TMT
Gawrilow et al. (2016)	RCT	23/23	8.3–13.6	Trampoline	VPA	5	-	-	Closed	Acute	Coloring in pictures	Go/No Go Task
Hattabi et al.(2019)	RTC	20/20	8–12	Aquatic games	MPA	90	2	12	Open	Chronic	Daily routine	Stroop Test;ROCF
Hung et al. (2016)	Crossover	34/34	8–12	Running	MVPA	30	-	-	Closed	Acute	Watching video	Task Switching Paradigm
Kadri et al.(2019)	RCT	20/20	(14.5 ± 3.5, 14.2 ± 3)	Taekwondo	NR	50	2	>12	Open	Chronic	Regular Physical Education	Stroop Test
Lee et al. (2017)	RCT	6/6	6–10	Combined exercise	MVPA	60	3	12	Closed	Chronic	NR	Stroop Test
Liang et al.(2022)	RCT	39/39	6–12	Combined exercise	MVPA	60	3	12	Open	Chronic	Daily routine	Flanker Task;The Tower of London test;TMT
Ludyga et al. (2017)	Crossover	16/18	11–16	Cycling & Coordinative	MPA	20	-	-	Open &Closed	Acute	Watching video	Flanker Task
Ludyga et al.(2022)	RCT	28/29	8–12	Judo	NA	60	2	12	Open	Chronic	Daily routine	Change Detection paradigm
Memarmoghaddam et al. (2016)	RCT	19/17	7–11	Aerobic exercise	MVPA	90	3	8	Open	Chronic	NR	Stroop Test;Go/No Go Task
Pan et al.(2016)	RCT	16/16	6–12	Table tennis	NR	70	2	12	Open	Chronic	NR	Stroop Test
Pan et al.(2019)	nRCT	15/15	7–12	Table tennis	NR	70	2	12	Open	Chronic	NR	Stroop Test;WCST
Piepmeier et al. (2015)	RCO	14/14	8–13	Cycling	MPA	30	-	-	Closed	Acute	Watching video	Stroop Test
Pontifex et al. (2013)	Crossover	20/20	8–10	Running	MPA	20	-	-	Closed	Acute	Reading	Flanker Task
Rezaei et al.(2018)	RCT	7/7	7–11	Yoga	NR	45	3	8	Closed	Chronic	NR	Letter–number sequencing micro test
Ruiter et al.(2022)	Crossover	18/18	15.62 ± 2.20	Cycling	LPA	30	-	-	Closed	Acute	Seated	Phonological Working Memory Task
Verret et al. (2012)	nRCT	10/11	7–12	PA programs	MVPA	45	3	10	Open	Chronic	NR	Walk/don’t walk pondered
Yu et al.(2020)	Crossover	24/24	8–12	Running	MPA	30	-	-	Closed	Acute	Watching video	Flanker Task

Note: NR = not report; CG = control group; IG = intervention group; WCST = Wisconsin Card Sorting Test; RCT = Randomized controlled trial; RCO = Randomized crossover; MPA = moderate physical activity; CSB = Color Span Backwards Task; ROCF = The Rey Osterrieth Complex Figure; TMT = rail Making Test.

### Risk of bias

**Fig 2:**
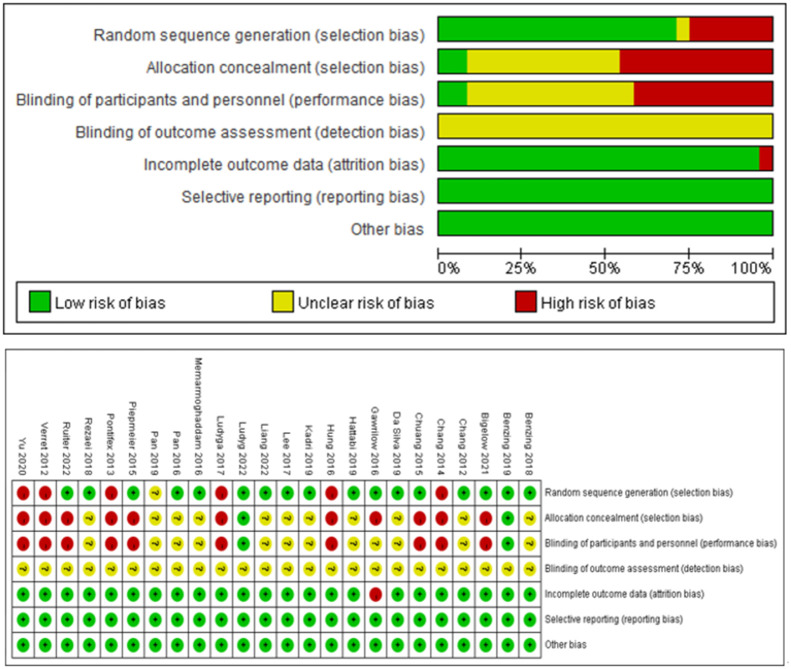
Risk of bias assessment for the included studies.

A total of 13 studies of RCTs [40–52], 2 studies of RCOs [36, 53], 3 studies of non-RCTs [35, 54, 55] and 6 studies of crossover trials [56–61] were included in this review. As seen in Fig 2, there was some bias in the included literature. The primary bias is that most studies did not state allocation concealment and did not describe whether double-blinding was performed on researchers and subjects.

### Sensitivity analysis

In studies with inhibitory control, working memory, and cognitive flexibility as outcome indicators, all P< 0.05 after excluding individual studies separately (see Table 3), the direction of the forest plot did not change essentially in any case, suggesting a low sensitivity of the data.

**Table 3:** Combined effect of executive function after excluding individual studies.

Task outcomes	N	SMD	I^2^/%	P (Combined effect)
Inhibitory control	19	-0.53~-0.42	54%~69%	<0.0001
Working memory	8	-0.57~-0.40	57%~68%	0.002~0.02
Cognitive flexibility	8	-0.53~-0.32	44%~76%	0.005~0.04

Notes. N = number of studies

### Publication bias

**Fig 3:**
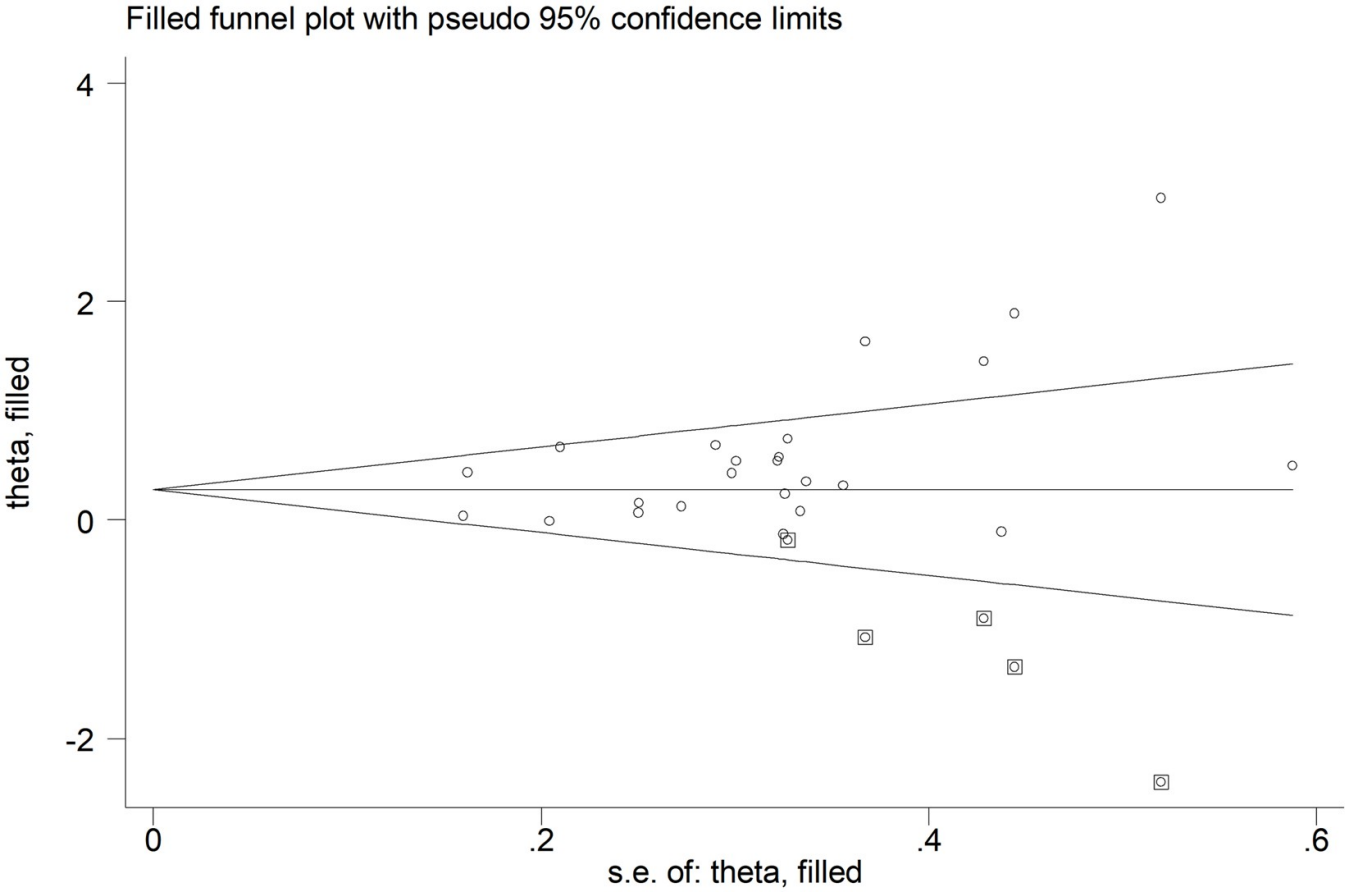
Funnel plot for visual inspection of publication bias.

The results of each of Egger’s tests are presented in Table 4. Publication bias tests were performed on the inhibitory control of outcome measures in the included studies. Egger’s test showed P = 0.012, suggesting potential publication bias in our included studies. The asymmetric funnel plot was corrected using the trim and fill algorithm. And the results are shown in Fig 3, which shows that publication bias could be eliminated after supplementing five studies, and the results did not change fundamentally, suggesting that the data results are relatively robust. Publication bias tests were conducted separately for the working memory and cognitive flexibility outcome indicators of the included studies, and none of the results showed the presence of publication bias in our included studies.

**Table 4:** Egger’s outcome for publication bias.

Task outcomes	N	Egger’s outcome	Publication bias
Inhibitory control	19	*P* = 0.012	Yes
Working memory	8	*p* = 0.300	No
Cognitive flexibility	8	*p* = 0.052	No

Notes. N = number of studies

### Summary of the meta-analysis results

#### The effect of physical activity on inhibitory control in ADHD children and adolescents

**Fig 4:**
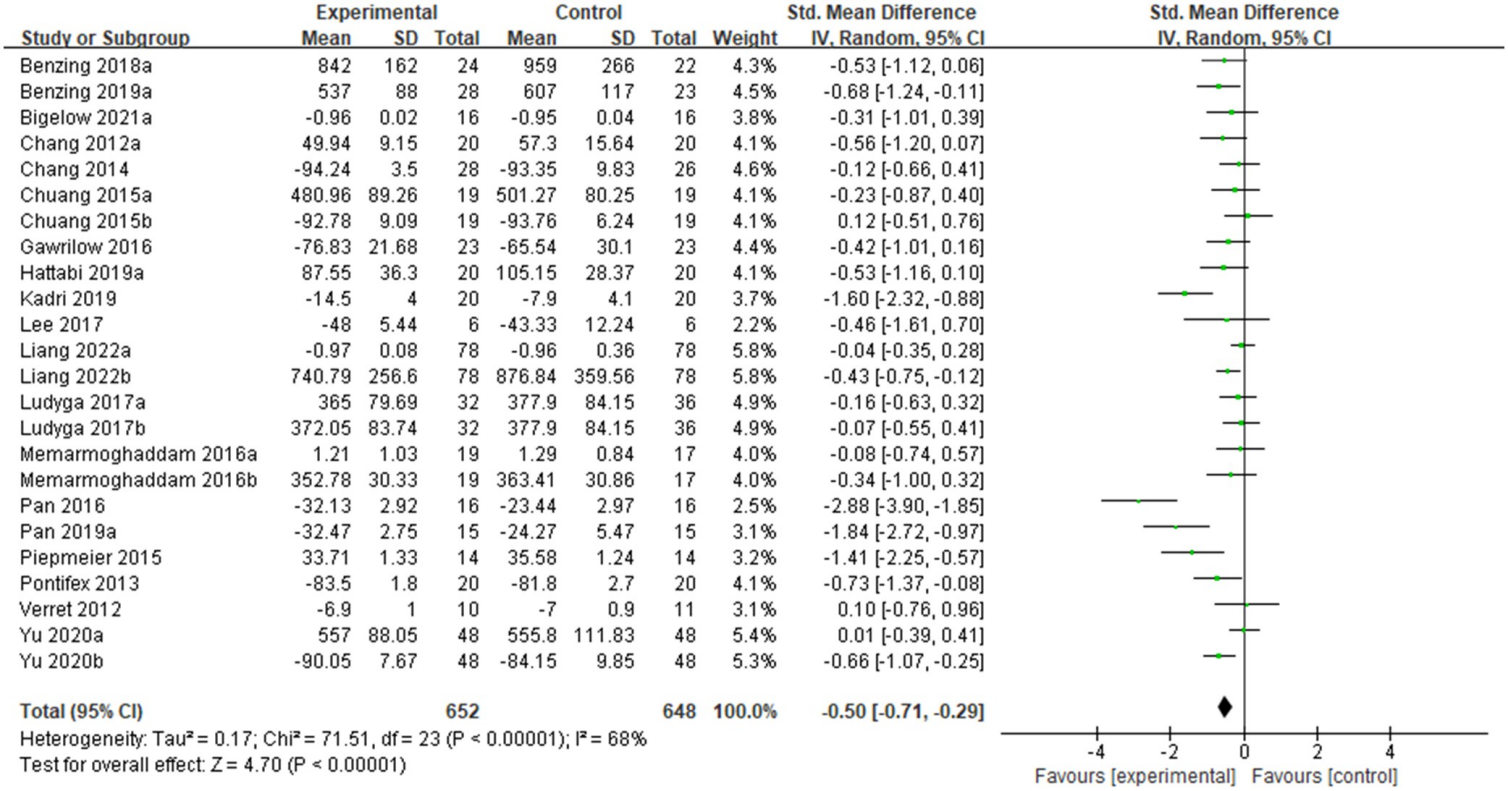
Forest plot of meta-analysis of the effect of PA interventions on inhibitory control in children and adolescents with ADHD.

As shown in Fig 4, 19 studies on the effects of PA interventions on inhibitory control in children and adolescents with ADHD were included. Testing showed significant heterogeneity (P < 0.00001, I^2^ = 68%), so a random-effects model was used for the analysis. The meta-analysis results showed a combined effect size of SMD = -0.50, 95% CI [-0.71, -0.29], P< 0.00001. The difference was statistically significant, indicating that exercise improves inhibitory control in children and adolescents with ADHD.

To explore the possible source of heterogeneity, the inhibitory control was analyzed in subgroups according to the intervention intensity, motor skills type, sessions of PA (Acute or Chronic), and amount of exercise per week (Frequency of PA interventions per week multiplied by the duration of each PA). Subgroup analyses suggest that the source of heterogeneity may not be related to the intensity of the intervention, type of motor skills, sessions of PA, and amount of exercise per week. In terms of the effect size, PA interventions based on moderate intensity, open motor skills, chronic, and lower weekly exercise amount produced higher effects on improving inhibitory control (see Table 5).

**Table 5:** Subgroup analysis of inhibitory control.

Overall	N	Effect size		Test result		Heterogeneity statistics			
		SMD	95% CI	Z	P	Q	d.f.	I^2^	P
**Intervention intensity**									
MPA	10	-0.43	[-0.68, -0.17]	3.32	0.0009	29.58	12	59	0.003
MVPA	6	-0.26	[-0.44, -0.09]	2.96	0.003	5.28	7	0	0.63
**Motor skills**									
Open	11	-0.60	[-0.94, -0.27]	3.54	0.0004	55.08	12	78	<0.00001
Closed	9	-0.39	[-0.62, -0.16]	3.31	0.0009	16.21	10	38	0.09
**Sessions of PA**									
Acute	9	-0.36	[-0.57, -0.16]	3.44	0.0006	17.77	11	38	0.09
Chronic	10	-0.66	[-1.04, -0.29]	3.45	0.0006	52.76	11	79	<0.00001
**Amount of exercise**									
Lower(<150min/week)	5	-1.34	[-2.23, -0.45]	2.96	0.003	25.68	4	84	<0.0001
Higher(>150min/week)	5	-0.25	[-0.42, -0.07]	2.71	0.007	4.45	6	0	0.62

#### The effect of physical activity on working memory in ADHD children and adolescents

**Fig 5:**
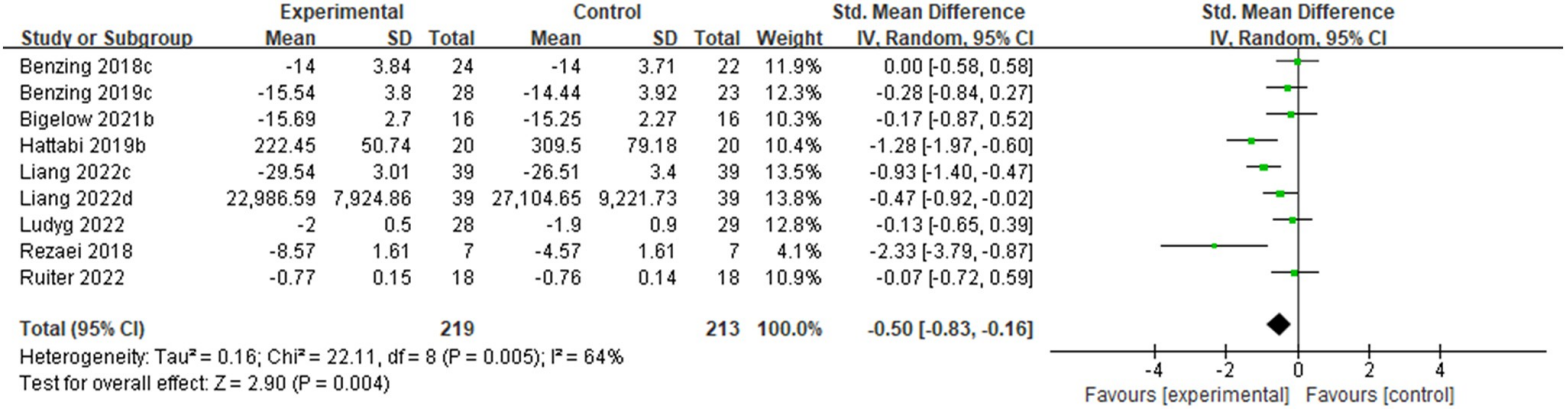
Forest plot of meta-analysis of the effect of PA interventions on working memory in children and adolescents with ADHD.

As shown in Fig 5., 8 studies on the effects of PA interventions on working memory in children and adolescents with ADHD were included. Testing showed significant heterogeneity (P = 0.005, I^2^ = 64%), so a random-effects model was used for the analysis. The meta-analysis results showed a combined effect size of SMD = -0.50, 95% CI [-0.83, -0.16], P = 0.004. The difference was statistically significant, indicating that exercise improves working memory in children and adolescents with ADHD. Subgroup analyses suggest that heterogeneity may be related to the type of motor skills, sessions of PA, and amount of exercise per week. Among them, in terms of the effect size, PA interventions based on open motor skills, chronic, and higher weekly exercise amounts produced higher effects on improving working memory(see Table 6).

**Table 6:** Subgroup analysis of working memory.

Overall	N	Effect size		Test result		Heterogeneity statistics			
		SMD	95% CI	Z	P	Q	d.f.	I^2^	P
**Intervention intensity**									
MPA	2	-0.73	[-1.82, 0.36]	1.31	0.19	4.97	1	80	0.03
MVPA	2	-0.49	[-0.99, 0.01]	1.92	0.05	6.14	2	67	0.05
**Motor skills**									
Open	5	-0.50	[-0.86, -0.14]	2.74	0.006	13.67	5	63	0.02
Closed	3	-0.63	[-1.63, 0.36]	1.25	0.21	8.00	2	75	0.02
**Sessions of PA**									
Acute	3	-0.07	[-0.44, 0.30]	0.37	0.71	0.14	2	0	0.93
Chronic	5	-0.72	[-1.15, -0.28]	3.25	0.001	15.74	5	68	0.008
**Amount of exercise**									
Lower(<150min/week)	3	-0.62	[-1.44, 0.21]	1.46	0.14	7.75	2	74	0.14
Higher(>150min/week)	2	-0.85	[-1.29, -0.41]	3.77	0.0002	4.22	2	53	0.0002

#### The effect of physical activity on cognitive flexibility in ADHD children and adolescents

**Fig 6:**
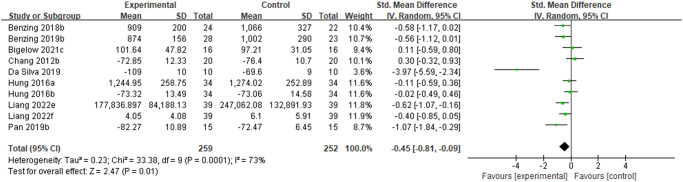
Forest plot of meta-analysis of the effect of PA interventions on cognitive flexibility in children and adolescents with ADHD.

As shown in Fig 6, 8 studies on the effects of PA interventions on cognitive flexibility in children and adolescents with ADHD were included. Testing showed significant heterogeneity (P = 0.0001, I^2^ = 73%), so the random-effects model was used for the analysis. The meta-analysis showed a combined effect size of SMD = -0.45, 95% CI [-0.81, -0.09], P = 0.01. The difference was statistically significant, indicating that exercise improves cognitive flexibility in children and adolescents with ADHD. Subgroup analyses suggest that the source of heterogeneity may be related to the intensity of the intervention, type of motor skills, and PA sessions. Among them, in terms of the effect size, PA interventions based on moderate to vigorous intensity, open motor skills, chronic, and lower weekly exercise amount produced higher effects on improving cognitive flexibility (see Table 7).

**Table 7:** Subgroup analysis of cognitive flexibility.

Overall	N	Effect size		Test result		Heterogeneity statistics			
		SMD	95% CI	Z	P	Q	d.f.	I^2^	P
**Intervention intensity**									
MPA	4	-0.10	[-0.49, 0.28]	0.52	0.60	8.63	4	54	0.07
MVPA	2	-0.52	[-0.80, -0.24]	3.64	0.0003	0.48	2	0	0.79
**Motor skills**									
Open	4	-0.58	[-0.82, -0.34]	4.76	<0.00001	2.17	4	0	0.70
Closed	4	-0.37	[-1.08, 0.35]	1.00	0.32	24.25	4	84	<0.0001
**Sessions of PA**									
Acute	4	-0.07	[-0.34, 0.19]	0.55	0.58	4.49	4	11	0.34
Chronic	4	-0.95	[-1.56, -0.34]	3.06	0.002	18.36	4	78	0.001
**Amount of exercise**									
Lower(<150min/week)	3	-1.63	[-3.02, -0.23]	2.29	0.02	15.14	2	87	0.0005
Higher(>150min/week)	1	-0.51	[-0.83,- 0.19]	3.11	0.002	0.44	1	0	0.51

## Discussion

This meta-analysis’s main purpose was to investigate PA interventions’ effects on core executive functions in children and adolescents with ADHD. The results showed that PA interventions were effective in enhancing inhibitory control, working memory, and cognitive flexibility in children with ADHD, suggesting that PA interventions are expected to be an effective alternative or adjunct to improve executive function in children and adolescents with ADHD.

The results of this meta-analysis are similar to the findings of the previous study, such as a systematic review of executive function as an outcome indicator of the effect of PA interventions in children with ADHD, which showed that PA interventions were effective in improving executive function in children with ADHD [62]. Furthermore, a meta-analysis showed that aerobic exercise and cognitively engaging PA could improve the executive function of children with ADHD [63]. Moreover, PA enhances executive function performance in children with ADHD, according to Welsch et al. [32]. Current research suggests that the mechanisms by which PA interventions improve executive function in children and adolescents with ADHD consist of two main aspects. On the one hand, the dysfunction of specific neural circuits in ADHD patients, such as frontoparietal, dorsal, and ventral attentional networks [64], and hyperarousal [65]. PA interventions can activate brain regions associated with executive function and increase functional connectivity between brain networks [66, 67], which can positively contribute to the improvement of executive function in children with ADHD. On the other hand, ADHD may be associated with an imbalance of catecholamine neurotransmitters [68]. Studies have found that PA increases the release of catecholamine neurotransmitters such as dopamine and norepinephrine [69, 70], which increases the level of arousal in the brain [71, 72], and thus promotes the development of executive functions in children with ADHD. In addition, PA interventions may improve cognitive function through modulation of the locus coeruleus–norepinephrine system [73].

This Meta-analysis explores moderating variables that may influence the effects of PA interventions on executive function in children and adolescents with ADHD. Subgroup analysis by PA intensity showed that MPA was most effective in improving inhibitory control in children and adolescents with ADHD. In contrast, MVPA was more beneficial for improving cognitive flexibility, thus suggesting that different PA intensities may affect executive function differently. For example, O’Malley et al. found that MPA was more effective than high-intensity and low-intensity PA in improving executive function after 13-week PA interventions in obese children aged 7 to 11 years [74]. According to a previous study, MPA was the most effective dose for improving cognitive and brain health [75]. Moreover, the arousal theory hypothesis suggests an inverted U-shaped relationship between exercise intensity and brain arousal levels. MPA corresponds to the optimal level of brain arousal and facilitates the efficient allocation of cognitive resources [76, 77]. Thus, MPA produces the best intervention effect on inhibitory control in children and adolescents with ADHD. However, this study showed that MVPA was more beneficial for cognitive flexibility in adolescents with children with ADHD, which may be because higher cognitive flexibility may require higher levels of arousal in the brain and the fact that higher-intensity PA can produce more brain-derived neurotrophic factors, resulting in better neuromodulatory effects of PA [78]. Currently, there are fewer studies on the effects of PA on working memory, and more research is needed to investigate the effects of different PA intensities on working memory in children and adolescents with ADHD.

Subgroup analysis showed that open motor skill-based PA effectively improved inhibitory control, working memory, and cognitive flexibility in children and adolescents with ADHD, whereas closed motor skill-based PA was not significant in improving working memory and cognitive flexibility. Closed motor skills are motor skills in which the environmental stimuli are relatively stable, and the motorist can stereotypically complete the movement according to his or her preplanned plan, such as running. In contrast, open motor skills require the motor subject to make adjustments to the movement according to the environmental stimuli during the completion of the movement, that is, the subject makes a dynamic response during the movement, such as table tennis [79]. Open motor skills are effective in improving executive function in children and adolescents with ADHD, probably because children are constantly judging external changes during the completion of movements. This process also requires the involvement of more attention and cognitive resources. In addition, physical activities such as taekwondo, which is based on open-motor skills, have specific coordination and cognitive demands which may contribute to the improvement of executive function in children with ADHD. However, in closed motor skills, the child only needs to rely on his or her proprioception to control his or her movements. The external environment and movements are consistent, which does not require many cognitive resources. Therefore, compared to closed motor skills, open motor skills may be more beneficial to improve the executive function of adolescents with ADHD. Furthermore, recent reviews have also concluded that open motor skills are most beneficial for improving cognitive function [80, 81].

The results of our meta-analysis showed that both acute and chronic PA interventions improved inhibitory control in children and adolescents with ADHD. In contrast, chronic PA interventions were required to improve working memory and cognitive flexibility. Furthermore, both lower and higher amounts of exercise improved inhibitory control and cognitive flexibility, while improved working memory required more exercise interventions. It can be seen that improvements in working memory in children and adolescents with ADHD may require more frequent and prolonged PA interventions than inhibitory control and cognitive flexibility. This may be because working memory, as an important central executive function, involves more brain areas such as the dorsolateral prefrontal lobe, inferior frontal gyrus, and cerebellum [82], more frequent and longer PA interventions to effectively increase the functional network connections between brain areas and promote the improvement of working memory in children. Secondly, it is also possible that the degree of impaired executive function and age differences in the subjects included in this study biased the results to some extent, which still needs to be further explored. In addition, the brain is more plastic in childhood, and chronic PA can cause changes in cerebral structure and function, increased angiogenesis and neurogenesis [83, 84], all of which are important factors in enhancing brain plasticity and executive function.

The strength of this meta-analysis is that it explores the effects of PA interventions on inhibitory control, working memory, and cognitive flexibility in children and adolescents with ADHD. Furthermore, this is the first meta-analysis to separately examine the effects of critical moderating variables of PA on three core executive functions in children and adolescents with ADHD. Nevertheless, the study also has some limitations. First, the small sample size of the included studies and the different ways of assessing outcome indicators may affect the statistical efficacy of the Meta-analysis. Besides PA’s beneficial and significant impact on the executive function of children and adolescents with ADHD, it is challenging to draw a firm conclusion. Secondly, only published studies in English were included, which may show some selection bias. Third, few studies detail how subjects, researchers, and outcome evaluators are blinded, and their random assignment is concealed. In addition, due to the small sample size and the inclusion of study participants who were mainly aged 8–12 years, no subgroup analysis of age was conducted in this study. Considering that age may also have an effect on the results, the effect of PA interventions on children with ADHD in different age groups needs to be further explored in the future.

## Conclusion

PA interventions effectively improved core executive function in children and adolescents with ADHD. MPA has the best effect on inhibitory control, and improving cognitive flexibility requires MVPA intervention; chronic and open motor skill-based PA interventions were more beneficial to children’s executive function, and improvements in working memory and cognitive flexibility required more frequent and longer PA interventions. It is recommended that future researchers focus on the intervention effects of PA on executive function in terms of the type, intensity, frequency, and time of PA in order to develop specific intervention programs to improve executive function in children and adolescents with ADHD.

## Supporting information

S1 Checklist PRISMA checklist. (PDF)
